# Evolution of the connection patterns of the cephalic lateral line canal system and its use to diagnose opsariichthyin cyprinid fishes (Teleostei, Cyprinidae)

**DOI:** 10.3897/zookeys.718.13574

**Published:** 2017-12-04

**Authors:** Taiki Ito, Toyoaki Fukuda, Toshihiko Morimune, Kazumi Hosoya

**Affiliations:** 1 Wetlands International Japan, 2F Jono Building II 17-1, Odenma-cho, Nihonbashi, Chuo-ku, Tokyo, 103-0011, Japan; 2 Tezukayama Junior & Senior High School, Gakuen-minami 3-1-3, Nara 631-0034, Japan; 3 Department of Environmental Management, Faculty of Agriculture, Kindai University, Nakamachi 3327-204, Nara 631-8505, Japan

**Keywords:** *Candidia*, heterochrony, morphology, *Parazacco*, sensory organs, *Zacco*

## Abstract

The cephalic lateral line canal systems were compared among 12 species of the cyprinid tribe Opsariichthyini. All species were characterized by the separation of the supraorbital canal from both the infraorbital and the temporal canals, and the left side of the supratemporal canal from the right side of the canal. In species of *Candidia*, *Opsariichthys*, *Parazacco*, and *Zacco*, and *Nipponocypris sieboldii* the temporal canal was separated from the preoperculomandibular canal. In *Nipponocypris temminckii* and *N. koreanus*, the temporal canal was connected to the preoperculomandibular canal. Separation of the left and right sides of the supratemporal canal is a possible synapomorphy of the opsariichthyin cyprinids. *Opsariichthys uncirostris* and *O. bidens* are unique among the opsariichthyins in that the connection between the infraorbital and temporal canals is retarded. The variation in arrangement of the cephalic lateral line canal system can be used as diagnostic characters for the opsariichthyin species.

## Introduction

The cyprinid tribe Opsariichthyini, of the subfamily Xenocypridinae (Liao et al. 2011; Kottelat 2013), comprises the East Asian genera *Opsariichthys* Bleeker, 1863, *Zacco* Jordan & Evermann, 1902, *Candidia* Jordan & Richardson, 1909, *Parazacco* Chen, 1982, and *Nipponocypris* Chen, Wu & Hsu, 2008 (Wang et al. 2007, Chen et al. 2008, Kottelat 2013). The opsariichthyins comprise approximately 19 species (Kim et al. 2005, Huynh and Chen 2013, Ito and Hosoya 2016). The opsariichthyin fishes are distributed in eastern Asia from Russia, Japan, through the Korean Peninsula to China, Taiwan, and northern Vietnam (Kottelat 2001, Kim and Park 2002, Chen and Chang 2005, Serov et al. 2006). They are loosely defined as a monophyletic group on the basis of a single character, namely, a long anal fin (Chen 1982), and recent molecular phylogenetic analyses support the monophyly of the group (e.g., Wang et al. 2007, Tang et al. 2013). However, morphological characters relevant for taxonomy have not been examined in detail for this group.

Variations in the connection pattern of the cephalic lateral line canals, and the number and the distribution of canal pores on the head have often been used in the study of interrelationships within the family Cyprinidae (Lekander 1949, Gosline 1975, Howes 1980, Chen et al. 1984, Hosoya 1986, Cavender and Coburn 1992, Arai and Kato 2003, Fujita and Hosoya 2005). Characteristics of the cephalic lateral line canal system have also been useful as diagnostic characters within the Cyprinidae (e.g., Illick 1956, Reno 1969, Gosline 1974, Kurawaka 1977). In particular, the connection pattern of the cephalic lateral line canal systems is species diagnostic in some cyprinid subfamilies such as the Acheilognathinae, Gobioninae, and Leuciscinae (Illick 1956, Kurawaka 1977, Arai and Kato 2003, Fujita and Hosoya 2005, Kawase and Hosoya 2015). However, the opsariichthyin cyprinids have not been thoroughly studied in terms of their cephalic lateral line canal system.

The objectives of the present study are to: (a) describe the connecting patterns of the cephalic lateral line canal system in the opsariichthyins, (b) provide diagnostic characters for the opsariichthyin species, (c) discuss the evolution of the connecting patterns observed.

## Materials and methods

The genus level classification of the Opsariichthyini follows Chen et al. (2008), although that classification still needs to be confirmed (Yin et al. 2015, cf. Hosoya 2013). The cephalic lateral line canal system was observed in 12 species of opsariichthyins; data on the canal system in the out-group were compiled from previous studies (Tables 1–2).

**Table 1.** Fish species used in the present molecular phylogenetic analysis.

**Classification**	**Species**	**Source**	**Accession no.**
Xenocypridinae			
opsariichthyin			
	*Candidia barbata*	Wang et al. (2007)	AY958200
	Candidia pingtungensis*^1^	Wang et al. (2007)	AY958201
	*Nipponocypris koreanus*	Chen et al. (2016b)	NC025286
	*Nipponocypris sieboldii*	Wang et al. (2007)	AY958198
	*Nipponocypris temminckii*	Wang et al. (2007)	AY958199
	*Opsariichthys bidens*	Wang et al. (2007)	AY958197
	Opsariichthys evolans*^2^	Wang et al. (2007)	AY968191
	Opsariichthys kaopingensis*^3^	Wang et al. (2007)	AY958189
	*Opsariichthys pachycephalus*	Wang et al. (2007)	AY958190
	*Opsariichthys uncirostris*	Wang et al. (2007)	AY958193
	*Parazacco spilurus*	Chang et al. (2016a)	NC023786
	*Zacco platypus*	Wang et al. (2007)	AY958194
others			
	*Culter alburnus*	unpublished	GU190362
	*Ctenopharyngodon idella*	Wang et al. (2008)	EU391390
	*Hemigrammocypris rasborella*	Tang et al. (2010)	AP011422
	*Hypophthalmichthys nobilis*	unpublished	EU343733
	*Ischikauia steenackeri*	He et al. (2004)	AF375862
	*Macrochirichthys macrochirus*	Tang et al. (2010)	AP011234
	*Metzia lineata*	Tang et al. (2010)	HM224305
	*Ochetobius elongatus*	He et al. (2004)	AF309506
	*Parachela siamensis*	Tang et al. (2010)	HM224300
	*Paralaubuca typus*	Saitoh et al. (2011)	AP011211
	*Squaliobarbus curriculus*	Tang et al. (2010)	HM224308
	Xenocypris macrolepis*^4^	Tang et al. (2010)	HM224310
Acheilognathinae			
	*Acheilognathus typus*	Saitoh et al. (2006)	AB239602
	*Rhodeus ocellatus*	Saitoh et al. (2006)	AB070205
	*Tanakia limbata*	Tang et al. (2010)	HM224309
Gobioninae			
	*Hemibarbus barbus*	Saitoh et al. (2006)	AB070241
	*Pseudorasbora parva*	Tang et al. (2010)	HM224302
Leuciscinae			
	*Scardinius erythrophthalmus*	unpublished	NC031561
	*Tribolodon hakonensis*	Imoto et al. (2013)	NC018820

*^1^ treated as *Candidia barbatus* (S); *^2^ *Zacco* sp. E; *^3^ *Z. pachycephalus* (S); *^4^ *Xenocypris argentea* by the authors.

**Table 2.** The connection states of the cephalic lateral line canal system in the opsariichthyins and out-group.

**Classification**	**Species**	**SO-IO**	**IO-TC**	**TC-POM**	**ST-ST**	**Source**
Xenocypridinae						
opsariichthyin						
	Candidia barbata*^1^	–	+	–	–	This study
	*Candidia pingtungensis*	–	+	–	–	This study
	*Nipponocypris koreanus*	–	+	+	–	This study
	*Nipponocypris sieboldii*	–	+	–	–	This study
	*Nipponocypris temminckii*	–	+	+	–	This study
	*Opsariichthys bidens*	–	±	–	–	This study
	*Opsariichthys evolans*	–	+	–	–	This study
	*Opsariichthys kaopingensis*	–	+	–	–	This study
	*Opsariichthys pachycephalus*	–	+	–	–	This study
	*Opsariichthys uncirostris*	–	±	–	–	This study
	Parazacco spilurus*^2^	–	+	–	–	This study
	*Zacco platypus*	–	+	–	–	This study
others						
	*Culter alburnus*	+	+	+	+	Takeuchi (2012)
	*Ctenopharyngodon idella*	+	+	+	+	Takeuchi (2012)
	*Hemigrammocypris rasborella*	–	+	–	+	Takeuchi et al. (2011)
	*Hypophthalmichthys nobilis*	–	+	+	+	Takeuchi (2012)
	*Ischikauia steenackeri*	+	+	+	+	Takeuchi (2012)
	*Macrochirichthys macrochirus*	+	+	+	†	Takeuchi (2012)
	*Metzia lineata*	–	+	+	+	Takeuchi (2012)
	*Ochetobius elongatus*	+	+	+	+	Takeuchi (2012)
	*Parachela siamensis*	+	+	+	†	Takeuchi (2012)
	*Paralaubuca typus*	+	+	+	+	Takeuchi (2012)
	*Squaliobarbus curriculus*	+	+	+	+	Takeuchi (2012)
	*Xenocypris macrolepis*	+	+	+	+	Takeuchi (2012)
Acheilognathinae						
	*Acheilognathus typus*	–	+	–	–	Arai and Kato (2003)
	*Rhodeus ocellatus*	–	+	–	–	Arai and Kato (2003)
	*Tanakia limbata*	–	+	–	–	Arai and Kato (2003)
Gobioninae						
	*Hemibarbus barbus*	+	+	+	+	Hosoya (1986)
	*Pseudorasbora parva*	–	+	–	+	Kawase and Hosoya (2015)
Leuciscinae						
	*Scardinius erythrophthalmus*	+	+	+	+	Takeuchi (2012)
	*Tribolodon hakonensis*	–	+	–	+	Kurawaka (1977)

IO, infraorbital canal; POM, preoperculomandibular canal; SO, supraorbital canal; ST, supratemporal canal; TC, temporal canal. SO-IO, continuity between the SO and IO; TC-IO, continuity between the TC and IO; TC-POM, continuity between the TC and POM; ST-ST, continuity between the left and right sides of the ST. Continuity (+), discontinuity (–), delay (±), and both sides of the ST connected and extending anteriorly (†). *^1^Three specimens had connected left and right sides of the ST. *^2^One specimen had a connected SO and IO.

**Figure 1.**
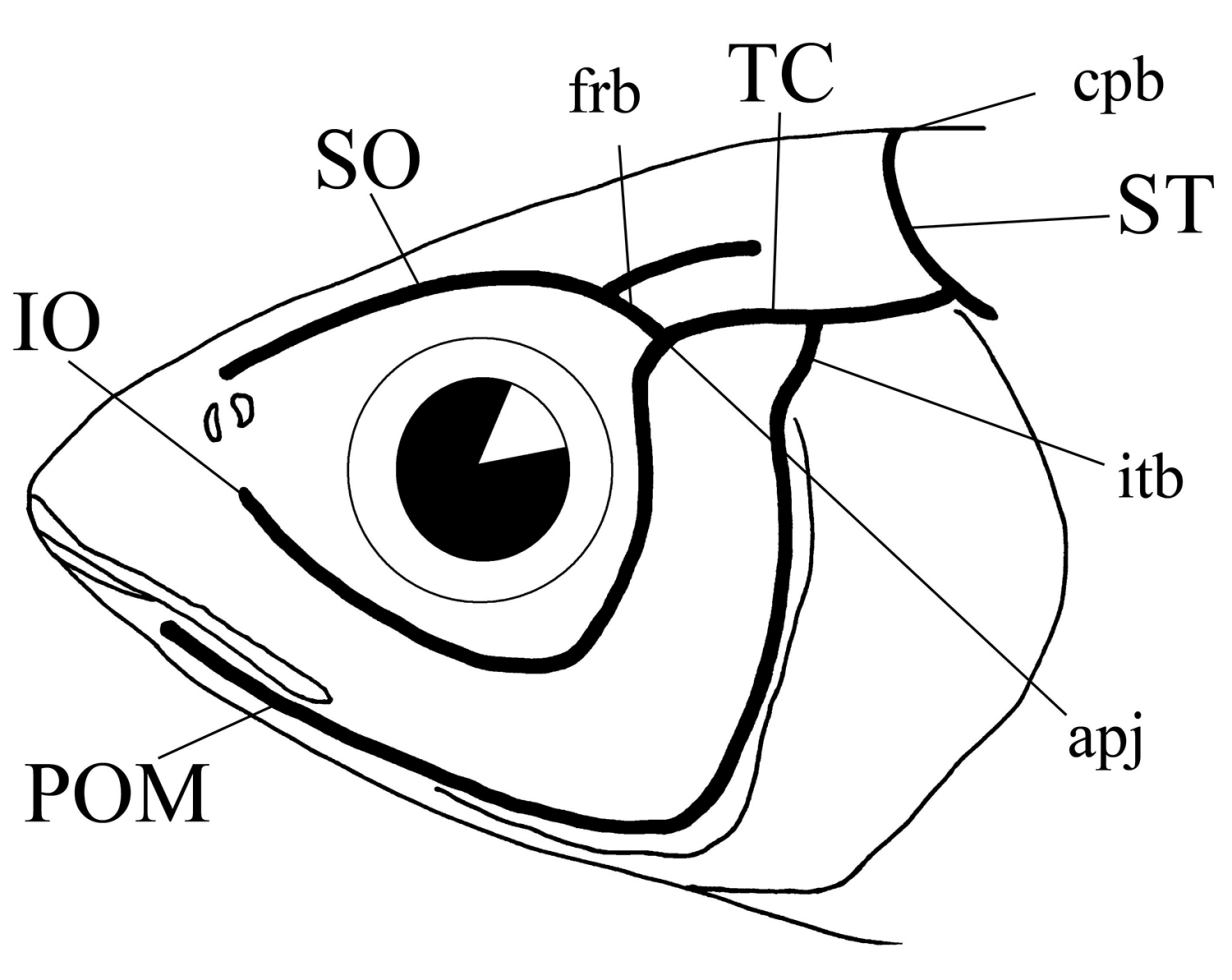
Terminology used for cephalic lateral line canal systems: **SO** supraorbital canal **IO** infraorbital canal **TC** temporal canal **POM** preoperculomandibular canal **ST** supratemporal canal **frb** frontal bridge **cpb** centroparietal bridge **itb** infratemporal bridge **apj** anteropteroitic joint.

Methods used for observation of the cephalic lateral line canal systems followed those of Fujita and Hosoya (2005). The canals were stained using Cyanine suminol 5R. The canal terminology follows that of Arai and Kato (2003), with additional reference to that of Fujita and Hosoya (2005). These are as follows: infraorbital canal (**IO**), preoperculomandibular canal (**POM**), supraorbital canal (**SO**), supratemporal canal (**ST**), and temporal canal (**TC**) (Fig. 1).

Furthermore, the canaliculi branching from each canal are defined as “bridges,” whereas the junctions connecting canals were termed “joints.” The three bridges and one joint were as follows: “frontal bridge” between SO and IO; “centroparietal bridge” recognizing that ST meets the opposite side ST; “infratemporal bridge” between POM and TC; and “anteropterotic joint” between IO and TC (Fig. 1).

In some species in cyprinid subfamilies such as Gobioninae and Leuciscinae, development of the cephalic lateral line canal system is generally completed when the fish is approximately 60 mm in total length (= TL) (Lekander 1949, Disler 1971, Hosoya 1986). Therefore, in the present study, specimens larger than 60 mm in TL were selected for examination. The pores on each canal were counted from end to end. Statistical tests were used to assess differences in the number of pores among the species. Tests for homogeneity of variance were carried out on the number of pores on each canal using Bartlett’s test in R 3.3.1 (R Core Team 2016). When the variances were homogeneous, the Tukey-Kramer test was used, whereas when variances were heterogeneous, the Steel-Dwass test in R 3. 3.1 was used.

To obtain a hypothesis about the branching pattern of the opsariichthyin species, we analyzed mitochondrial cytochrome *b* (cyt *b*) gene sequences downloaded from GenBank. This is because molecular data for the cyt *b* gene sequence of all the species examined in the present study have been accumulated by previous studies (Table 1). Cyt *b* sequence alignment of 1137 bp long sequences was performed using MEGA 7 (Kumar et al. 2016) and checked manually for accuracy. Maximum likelihood (ML) analysis for phylogenetic reconstruction was applied using PAUP* v. 4.0b10 (Swofford 2002). Models of molecular evolution were selected using the program MODELTEST v.3.7 (Posada and Crandall 1998), with the best fitting model being determined by the Akaike information criteria (AIC) (= GTR+G+I model, in the present analysis). Three species of the subfamily Acheilognathinae, two species of the subfamily Gobioninae, two species of the subfamily Leuciscinae, and 12 species of the Xenocypridinae were chosen as out-groups (Table 1). Polarity in the character evolutions of the connecting pattern of the cephalic lateral line canals was determined by character state reconstruction using Mesquite v.2.75 (Maddison and Maddison 2010) with maximum parsimony methodology. Maximum parsimony character state reconstruction was performed on the ML tree.

Specimens studied are deposited in the following institutions: Chonbuk National University, Jeollabuk-do, Korea (**CNUC**); Department of Fisheries, Faculty of Agriculture, Kyoto University, Kyoto, Japan (**FAKU**); Fisheries Research Laboratory, Mie University, Mie, Japan (**FRLM**); Lake Biwa Museum, Shiga, Japan (LBM); the National Museum of Nature and Science, Tsukuba, Japan (**NSMT**); Swedish Museum of Natural History, Stockholm, Sweden (**NRM**); Smithsonian Institution National Museum of Natural History, Washington DC, United States (**USNM**). The institutional code of the Faculty of Agriculture, Kindai University, was changed from **FKUN** (Department of Fisheries, Kindai University, Nara) to **KUN-P** (Kindai University, Nara, Pisces) with faculty reorganization in 2005.

### Material examined

*Candidia barbata* (Regan, 1908): FKUN 34180, 1, 94.8 mm standard length (= SL), Tamsui River, Taipei, Taiwan; FKUN 35264–35272, 9 , 49.3–94.8 mm SL, Shueili River, Nantou, Taiwan; KUN-P 44430–44433, 4 , 94.7–103.0 mm SL, Houlong River, Miaoli, Taiwan.

*Candidia pingtungensis* Chen Wu & Hsu, 2008: FKUN 35214–35215, KUN-P 44492, 44515–44516, 5, 53.3–112.9 mm SL, Kaoping River, Pingtung, Taiwan.

*Nipponocypris koreanus* (Kim, Oh & Hosoya, 2005): KUN-P 40584–40591, 8, 69.3–111.9 mm SL, Nakdong River, Yeongwol, Korea; KUN-P 44463, 44475–44476, 3, 111.3–137.2 mm SL, Nakdong River, Gyongnam, Korea.

*Nipponocypris sieboldii* (Temminck & Schlegel, 1846): KUN-P 40564–40573, 10, 81.3–105.0 mm SL, Yamato River Nara Pref., Japan; KUN-P 44764–44767, 4, 63.7–85.8 mm SL, Kizu River, Kyoto Pref., Japan.

*Nipponocypris temminckii* (Temminck & Schlegel, 1846): KUN-P 40574–40581, 40583, 9, 85.2–100.9 mm SL, Kizu River, Kyoto Pref., Japan; KUN-P 45003, 45005–45006, 3, 79.1–145.3 mm SL, Shiomi River, Saga Pref., Japan; KUN-P 45104–45105, 45109, 3, 110.8–130.9 mm SL, Kawatana River, Nagasaki Pref., Japan.

*Opsariichthys bidens* Günther, 1873: LBM 8852, 47588, FRLM 28191–28192 (captive bred individuals), USNM 86307, 5, 66.7–108.1 mm SL, ChangJiang River, Sichuan, China; NSMT 12464, 10, 61.7–80.5 mm SL, Cheng-te, Hebei, China.

*Opsariichthys evolans* (Jordan & Evermann, 1902): FKUN 35196–35199, 35255, 35256, 6, 50.9–81.1 mm SL, Fengshan River, Hsinchu, Taiwan; KUN-P 44427–44429, 3, 69.5–80.6 mm SL, Houlong River, Miaoli, Taiwan.

*Opsariichthys kaopingensis* Chen, Wu & Huang, 2009: KUN-P 40545–40547, 44402, 44404–44405, 44407, 7, 69.2–83.0 mm SL, Kaoping River, Pingtung, Taiwan.

*Opsariichthys pachycephalus* (Günther,1868): FKUN 35179–35183, 35194, 35195, 7, 69.4–95.4 mm SL, Fengshan River, Hsinchu, Taiwan; FKUN 35245, 35250, 35252, 3, 56.0–70.3 mm SL, Keelung River, Taipei, Taiwan.

*Opsariichthys uncirostris* (Temminck & Schlegel, 1846): FKUN 16487–16488, 16492, 16495, 4, 211.5–228.0 mm SL, Ishida River, Shiga Pref., Japan; FKUN 16561, 16569, 16574, 3, 83.9–139.6 mm SL, Lake Biwa, Shiga Pref., Japan; KUN-P 40548–40554, 40592, 44528, 44529, 10, 145.1–231.8 mm SL, Mano River, Shiga Pref., Japan; FKUN 31878–31880, 3, 65.8–80.7 mm SL, Bukhan River, Korea; KUN-P 40636, 1, 206.5 mm SL, Gupo fish market, Korea; CNUC 37632, 1, 213.1 mm SL, Mangyeong River, Korea.

*Parazacco spilurus* (Günther, 1868): NRM 59489, 2, 56.6–82.8 mm SL, Pearl River, Guangxi Province, China; KUN-P 44899, 45852, 2, 57.5–105.6 mm SL, Pearl River, Hongkong, China.

*Zacco platypus* (Temminck & Schlegel, 1846): KUN-P 40555–40563, 9, 79.1–93.0 mm SL, Yamato River, Nara Pref., Japan; KUN-P 44379, 44381, 44383, 44386–44388, 6, 114.5–123.4 mm SL, Mono River, Shiga Pref., Japan.

## Results

**Figure 2.**
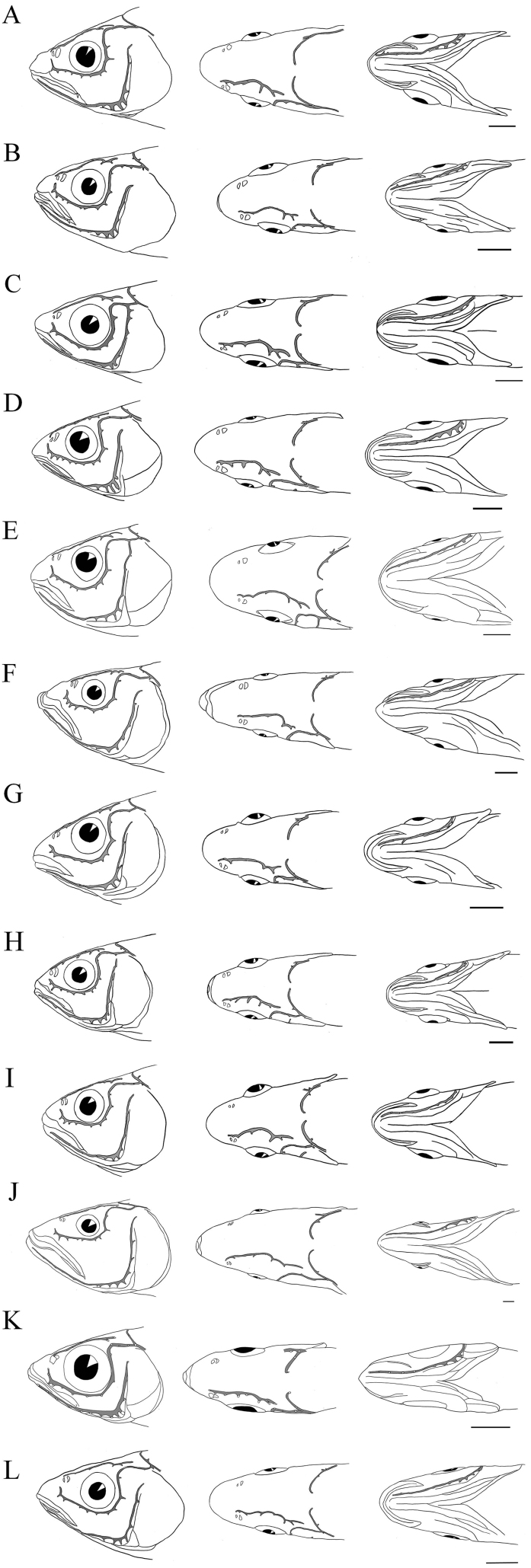
Diagram of the cephalic lateral line canal systems in the opsariichthyin fishes. **A** *Candidia barbata*, FKUN 34180, 94.8 mm SL **B** *C. pingtungensis*, FKUN 35215, 72.9 mm SL **C** *Nipponocypris koreanus*, FKUN 40587, 94.1 mm SL. Scale bar 5 mm. **D** *N. sieboldii*, FKUN 40571, 90.5 mm SL **E** *N. temminckii*, FKUN 40575, 94.5 mm SL **F** *Opsariichthys bidens*, LBM 8852, 94.8 mm SL. Scale bar 5 mm. **G** *O. evolans*, FKUN 35199, 81.1 mm SL **H** *O. kaopingensis*, KUN-P40545, 80.0 mm SL **I** *O. pachycephalus*, FKUN 35181, 69.4 mm SL. Scale bar 5 mm. **J** *O. uncirostris*, FKUN 16487, 219.0 mm SL **K** *Parazacco spilurus*, KUN-P45852, 57.5 mm SL **L** *Zacco platypus*, FKUN 40558, 93.0 mm SL. Scale bar 5 mm.

The cephalic lateral line canal system is comprised of five canals, three bridges, and one joint in all opsariichthyin specimens examined (Fig. 2A–L). No intraspecific variation was found in the connection patterns of the cephalic lateral line canals when conspecific specimens of similar size were compared.

The canals were usually well ossified, although part of the POM (see below), the frontal bridge, the infratemporal bridge, and the anteropterotic joint were cutaneous tubes. The SO was housed in the nasal and frontal bones. This canal was separated from the IO and TC in all the opsariichthyin fishes (with the exception of one specimen of *P. spilurus* in which the SO and IO were connected: NRM 59489, 82.8 mm SL).The IO runs along a series of five infraorbital bones. This canal was connected with the TC in all species; however, the canal was separated from the TC in individuals less than ca. 180 mm SL in *O. uncirostris* and ca. 100 mm SL in *O. bidens*. The POM was found in the anguloarticular, dentary, and preopercular bones. In the anguloarticular, the canal was cutaneous. The TC runs in the pterotic. No connection between the TC and POM was observed, except in *N. temminckii* and *N. koreanus*, in which the TC was connected with the POM by the infratemporal bridge. The ST passes through the parietal bone. In all the opsariichthyin species, the left and right sides of the ST were typically separated (except for three specimens of *C. barbata* in which left and right sides of the ST connected: FKUN 35270–35272, 49.3–54.7 mm SL). The ST was connected with the TC and the trunk canal in all the opsariichthyin species.

Connecting patterns of the cephalic lateral line canal system of the out-groups are shown in Table 2.

The number of pores on each canal are shown in Table 3. The opsariichthyins had 8–9 pores on the SO; 10–14 pores on the IO, 3–5 pores on the TC; 12–17 pores on the POM; 2–3 pores on the ST. The number of pores on the POM differs significantly between *O. uncirostris* and *O. pachycephalus*, *O. evolans*, *Z. platypus* (*P* < 0.01), *O. kaopingensis* and *N. sieboldii* (*P* < 0.05); between *O. bidens* and *O. evolans* (*P* < 0.05) and *Z. platypus* (*P* < 0.01); between *N. koreanus* and *O. pachycephalus*, *O. evolans*, and *Z. platypus* (*P* < 0.01); between *N. temminckii* and *O. evolans*, *Z. platypus* (*P* < 0.01) and *O. pachycephalus* (*P* < 0.05); and between *C. barbata* and *Z. platypus* (*P* < 0.05). No significant difference was found in the number of pores on the IO, SO, TC, and ST among the opsariichthyin fishes.

**Table 3.** Mode, average ± standard deviation, and range of the number of pores in each part of the cephalic lateral line canal in the opsariichthyin cyprinids.

**Species**	**SO**	**IO**	**TC**	**POM**	**ST**
*Candidia barbata*	8, 8.00 ± 0, 8	12, 11.91 ± 0.30, 11–12	4, 4.00 ± 0, 4	14, 14.27 ± 1.27, 12–16	3, 3.00 ± 0, 3
*Candidia pingtungensis*	8, 8.20 ± 0.45, 8–9	12, 12.60 ± 0.89, 12–14	4, 4.00 ± 0, 4	15, 14.20 ± 0.84, 13–15	3, 2.80 ± 0.45, 2–3
*Nipponocypris koreanus*	8, 8.00 ± 0, 8	12, 11.82 ± 0.60, 11–13	4, 3.91 ± 0.30, 3–4	15, 14.91 ± 0.83, 13–16	3, 3.00 ± 0, 3
*Nipponocypris sieboldii*	8, 8.00 ± 0, 8	12, 11.93 ± 0.83, 10–13	4, 4.11 ± 0.31, 4–5	14, 13.79 ± 0.97, 12–15	3, 3.00 ± 0, 3
*Nipponocypris temminckii*	8, 8.00 ± 0, 8	12, 11.67 ± 0.49, 11–12	4, 4.07 ± 0.26, 4–5	15, 14.53 ± 0.99, 13–17	3, 3.00 ± 0, 3
*Opsariichthys bidens*	8, 8.00 ± 0, 8	12, 12 ± 0.37, 11-13	4, 4 ± 0.37, 3-5	14, 14.33 ± 0.70, 13–16	3, 3.00 ± 0, 3
*Opsariichthys evolans*	8, 8.00 ± 0, 8	12, 11.56 ± 0.73, 10–12	4, 4.00 ± 0, 4	12, 13.00 ± 0.87, 12–14	3, 3.00 ± 0, 3
*Opsariichthys kaopingensis*	8, 8.00 ± 0, 8	12, 11.57 ± 0.53, 11–12	4, 4.00 ± 0, 4	13, 13.43 ± 0.79, 13–15	3, 3.00 ± 0, 3
*Opsariichthys pachycephalus*	8, 8.00 ± 0, 8	12, 12.22 ± 0.67, 11–13	4, 4.11 ± 0.33, 4–5	14, 13.22 ± 0.83, 12–14	3, 3.00 ± 0, 3
*Opsariichthys uncirostris*	8, 8.05 ± 0.22, 8–9	12, 11.95 ± 0.51, 11–13	4, 4.05 ± 0.22, 4–5	14, 14.95 ± 1.10, 14–17	3, 3.00 ± 0, 3
*Parazacco spilurus*	8, 8.00 ± 0, 8	11, 11.25 ± 0.50, 11–12	4, 4.00 ± 0.82, 3–5	14, 13.50 ± 1.00, 12–14	3, 3.00 ± 0, 3
*Zacco platypus*	8, 8.07 ± 0.27, 8–9	12, 11.79 ± 0.43, 11–12	4, 4.00 ± 0, 4	13, 13.00 ± 0.55, 12–14	3, 3.00 ± 0, 3

IO, infraorbital canal; POM, preoperculomandibular canal; SO, supraorbital canal; ST, supratemporal canal; TC, temporal canal. When both sides of the ST canal were connected to form a single pore on the pariental, the numbers shown include this pore.

**Figure 3.**
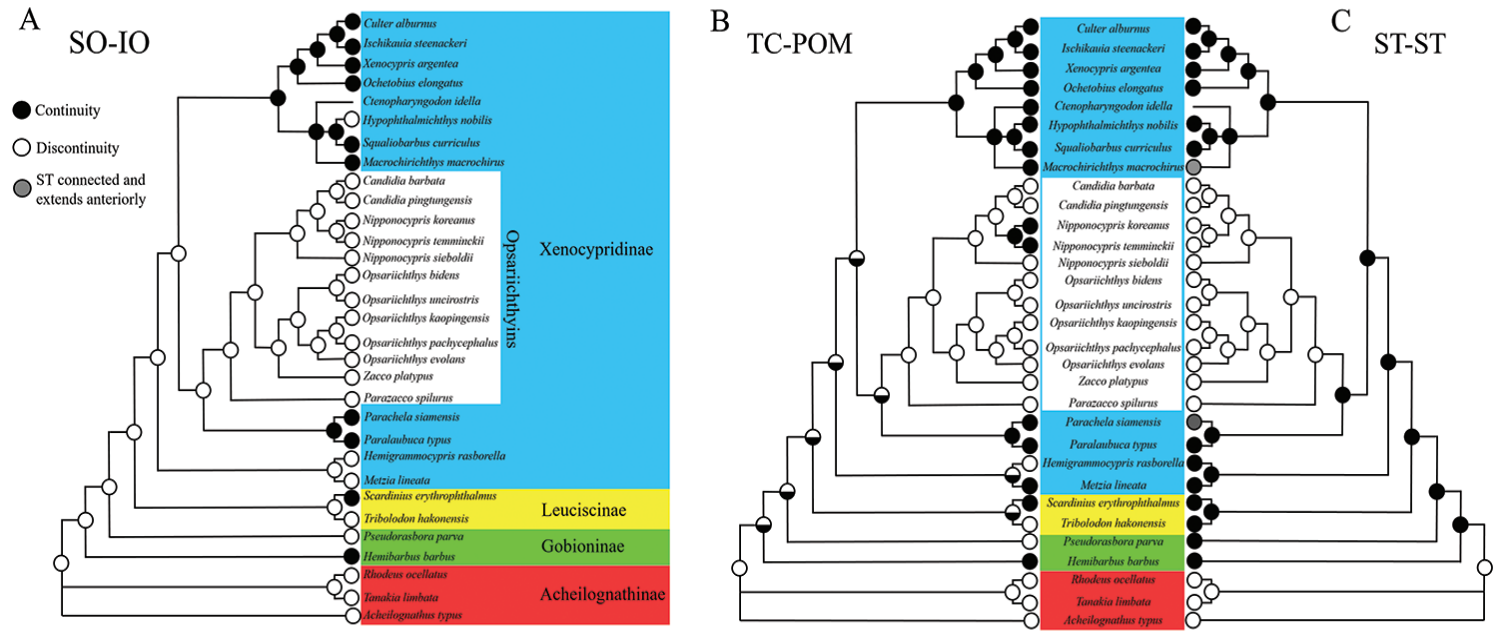
Parsimonious ancestral state reconstruction of the connecting states of the cephalic lateral line canal systems of the opsariichthyin fishes and their out-groups from the maximum likelihood tree inferred from cytochrome *b* sequences (InL = 12054.39). **A** The connecting states between the supraorbital (**SO**) and infraorbital (**IO**) canals **B** the connecting states between the temporal (**TC**) and preoperculomandibular (**POM**) canals **C** the connecting states between the left and right sides of the supratemporal canals (**ST**). The color of each node indicates the connecting states of the cephalic lateral line canal system: black, continuity; white, discontinuity; gray, both sides of the **ST** connected and extending anteriorly.

The topology of the ML tree is shown Figure 3. The ancestor at the root of the opsariichthyins on the ML tree was reconstructed as having canal separation between the SO and IO (Fig. 3A). The canal connection between the SO and IO was estimated to have occurred in at least four independent lineages in the out-group (see Fig. 3A). The ancestor at the root of the opsariichthyins was reconstructed as having canal separation between the TC and POM. In the opsariichthyins, the canal connection between the TC and POM emerged in the ancestor of *N. temminckii* and *N. koreanus* (Fig. 3B). The canal connection between the TC and POM emerged at least five lineages in the out-groups (Fig. 3B). The canal separation between the left and right sides of the ST independently emerged twice in the ancestors of the Acheilognathinae and the opsariichthyin (Fig. 3C). The canal connection and anterior extension between the right and left of the ST occurred at least twice in the out-groups (see Fig. 3C).

## Discussion

### The cephalic lateral line canal systems as a diagnostic character

Significant differences were found in the number of pores on the POM among some opsariichthyin species. However, the number of pores on these canals was found to vary within each species, and there was an overlap of ranges among all observed species (Table 3). Therefore, the number of pores on the cephalic lateral line canals does not provide reliable diagnostic character states for the opsariichthyin species.

In contrast, the connecting pattern of the cephalic lateral line canals provides useful diagnostic character states for some species of the opsariichthyins. *Nipponocypris temminckii* and *N. koreanus* are clearly distinguished from the very similar species *N. sieboldii* by the connection between the POM and TC through the infratemporal bridge. Similarly, *O. uncirostris* can be distinguished from *O. bidens* on the basis that the two species have different sizes at which the connection between the IO and TC attains completion (ca. 180 mm SL vs. ca. 100 mm SL, respectively), although many investigators have indicated that these two species can only be distinguished by the number of scales in the lateral series (e.g., Bănărescu 1968, Chen 1982).

### Character evolution

All opsariichthyin species share the canal separation between the left and right sides of the ST. Although, this character state also occurs at the root of the Acheilognathinae, this characteristic strongly supports the monophyly of the opsariichthyins, because the characteristic was derived only once from the common ancestor of the opsariichthyins in the Xenocypridinae. The opsariichthyins have been defined in terms of a single shared character state, viz. a long anal fin (Chen 1982). Based on our analysis, the canal separation between the left and right sides of the ST is suggested as a possible synapomorphy of the opsariichthyin by the character state reconstruction. In addition, in the opsariichthyins, the canal connection between the POM and TC emerged in the ancestor of *N. temminckii* and *N. koreanus* (Fig. 3). The canal connection between the POM and TC is a possible synapomorphy of *N. temminckii* and *N. koreanus*. In the present study, there was no synapomorphy to link *N. temminckii* and *N. koreanus*, and *N. sieboldii*, and the current recognized genus *Nipponocypris* is not monophyletic. Our analyses suggested that *Nipponocypris* is paraphyletic, but further taxonomic study is required.

### Evolution of the cephalic lateral line canal system ontogeny in *Opsariichthys uncirostris* and *O. bidens*

*Opsariichthys uncirostris* and *O. bidens* have a unique ontogeny of the cephalic lateral line canal system. In the Cyprinidae, the cephalic lateral line canal systems are generally completed at 40–60 mm in TL (Lekander 1949, Disler 1971, Hosoya 1986). In the opsariichthyins (with the exception of *O. uncirostris* and *O. bidens*), they are completed by approximately 60 mm SL. In *O. uncirostris* and *O. bidens*, canalization of the IO and TC through the anteropterotic joint is delayed until the individual reaches a mature size. Retardation of cephalic lateral line formation in both species can be explained in as a form of “isomorphosis”, a term proposed by Reilly et al. (1997) for cases in which heterochrony does not affect the offset shape. This is exemplified by a character state that is identical in the ancestor and descendant, although the descendant arrives at the same shape via a different ontogenetic trajectory. The delayed offset of cephalic lateral line formation seen in *O. uncirostris* and *O. bidens* is identical to “hypermorphosis” (sensu Reilly et al. 1997; cf. Hanken 2015), and the retardation of its developmental rate is identical to “deceleration” (sensu Reilly et al. 1997; cf. Hanken 2015). Both species are unique among opsariichthyin fishes in that they grow to between 250 (*O. bidens*) and 300 (*O. uncirostris*) mm TL (other opsariichthyin species are typically < 200 mm TL), and thus require more time to reach their mature size than other opsariichthyin species (Nakamura 1969, Tanaka 1970, Xing et al. 2007, Sui et al. 2012). Therefore, the retardation of cephalic lateral line formation in both species may be attributable to prolongation of the immature stage.
